# The Discovery of Modern Surgical Anesthesia

**Published:** 1893-11

**Authors:** 


### *EDITORIAL.*

The office of THE JOURNAL is in room 330 Equitable Building.

Address all communications, and make all remittances payable to THE ATLANTA MEDICAL AND SURGICAL JOURNAL, Atlanta, Ga.

Articles for publication should reach this office not later than the 15th instant.

The editors are not responsible for views expressed by contributors.

*In the language of an esteemed contemporary, we wish again to remind our subscribers that now is the time to pay up.* THE JOURNAL *"expects every man to do his duty."*

### THE DISCOVERY OF MODERN SURGICAL ANESTHESIA.

The *Virginia Medical Monthly* for October contained an article by the editor of this JOURNAL entitled, "A Contribution to the History of the Discovery of Modern Surgical Anesthesia." The object of the communication was to place before the profession some new data relative to the claim of the late Dr. Crawford W. Long of Athens, Ga., as the original discoverer of the anesthetic property of sulphuric ether. These data pertained mainly to the alleged relations existing at the time of the discovery (1841–42) between Dr. Long and a young man, P. A. Wilhite. This Wilhite afterwards became a practicing physician (at Anderson, S. C.), and it was through information received from him in 1876 that Dr. J. Marion Sims presented the case of Dr. Long to the profession (*Virginia Medical Monthly*, May, 1877). The paper of Dr. Sims was very unsatisfactory to Dr. Long. It contained many errors, the principal one relating to Wilhite himself.

Sims's article makes it appear that Wilhite and three other young men were students in Dr. Long's office prior to the events of 1842; that Wilhite related to Dr. Long how he had playfully etherized a negro boy in 1839, and that Dr. Long was encouraged from this story to believe that ether might be used to prevent pain in surgical operations.

Our own paper was prepared from certificates, correspondence and other data which had been gathered by Dr. Long in support of his claim, and which were kindly placed in our hands by the Long family. Some of these papers are from Dr. Wilhite himself. One of them certifies that he entered the office of Dr. Long in October, 1844, and that he heard Dr. Long speak of having used ether to prevent pain in surgical operations, etc. As to the negro boy incident, Dr. Long appears to have known nothing about it until nearly forty years after it is said to have occurred, and then it was related to him for the first time by Dr. Wilhite.

From the evidence before us we were sure that Dr. Wilhite had achieved a prominence in connection with this matter which he did not deserve. His own letters to Dr. Long (which Dr. Sims never saw) relieve him from all possibility of credit in this connection. One of them (January 16, 1877) urges Dr. Long to furnish Dr. Sims with certain information in order that he (Long) "might, and justly too, receive the credit of this great discovery." Wilhite told Sims in 1876 that "Long was the real and original discoverer of anesthesia, and believed he would be so acknowledged if all the facts in the case were fully set forth." So, from all the evidence, including Wilhite's own testimony, it becomes certain that he could not have furnished Dr. Long with any assistance or suggestion whatever.

Dr. Long himself stated that he received his first intimation of the anesthetic possibilities of ether from observing a number of young men inhaling it in sport. Several of them received small injuries, but felt no pain until the effect of the ether began to pass off. His first opportunity to test the ether in surgery came March 30, 1842, when he removed a small tumor from the neck of James Venable. Other operations followed somewhat slowly, and Dr. Long waited for a case of capital surgery before publishing his results already obtained. Then it was that his New England contemporaries were making their observations. Their first case was operated upon October 16, 1846, and it took them just eleven days to *patent* their ether, and then publish to the world their great "invention."

Thus it happened that the credit of this discovery, the "priceless gift of applied anesthesia," has been bestowed upon certain parties while belonging properly to another. But the profession has begun to recognize the merits of Dr. Long's case, and the time is almost at hand in our medical history when he will occupy the position which he eminently deserves, as the real and unassisted discoverer of anesthesia.

